# Questions of Interest to the Dental Specialist

**Published:** 1883-01

**Authors:** 


Questions of Interest to the Dental Specialist.—We shall be glad to receive questions upon matters of general interest to the dental profession, and will to the best of our ability answer such through the columns of the Independent Practitioner. Incidentally we would mention that to be sure of obtaining the benefit of such answers, if benefits there be, one should be a subscriber. Send in your name to the publishers, and we pledge our best efforts to give you the worth of your money.

